# Fast-scan Cyclic Voltammetry for the Characterization of Rapid Adenosine Release

**DOI:** 10.1016/j.csbj.2014.12.006

**Published:** 2014-12-29

**Authors:** Michael D. Nguyen, B. Jill Venton

**Affiliations:** Department of Chemistry, University of Virginia, McCormick Road, PO BOX 400319, Charlottesville, VA 22904, United States

**Keywords:** Adenosine, Electrochemistry, Microelectrodes, Purine signaling, Biosensors

## Abstract

Adenosine is a signaling molecule and downstream product of ATP that acts as a neuromodulator. Adenosine regulates physiological processes, such as neurotransmission and blood flow, on a time scale of minutes to hours. Recent developments in electrochemical techniques, including fast-scan cyclic voltammetry (FSCV), have allowed direct detection of adenosine with sub-second temporal resolution. FSCV studies have revealed a novel mode of rapid signaling that lasts only a few seconds. This rapid release of adenosine can be evoked by electrical or mechanical stimulations or it can be observed spontaneously without stimulation. Adenosine signaling on this time scale is activity dependent; however, the mode of release is not fully understood. Rapid adenosine release modulates oxygen levels and evoked dopamine release, indicating that adenosine may have a rapid modulatory role. In this review, we outline how FSCV can be used to detect adenosine release, compare FSCV with other techniques used to measure adenosine, and present an overview of adenosine signaling that has been characterized using FSCV. These studies point to a rapid mode of adenosine modulation, whose mechanism and function will continue to be characterized in the future.

## 1 Introduction

Adenosine signaling was first discovered in 1929 and it regulates numerous physiological processes at the cellular level. Adenosine modulates sleep [1], breathing [2], heart rate [3], blood flow [4], and neurotransmission [5]. Adenosine is considered a neuromodulator, since it acts over extended periods of time and over a diffuse area [6]. Adenosine is also neuroprotective and released during events such as stroke and ischemia [7]. A breakdown product of ATP, adenosine can build up due to energy consumption and metabolic processes [6]. The effects of adenosine are complicated because it can be both excitatory and inhibitory. Pharmacological studies have demonstrated that A_1_ receptors primarily mediate inhibitory effects while A_2a_ receptors facilitate excitatory effects of adenosine [8]. For example, the A_1_ adenosine receptor modulated preconditioning against anoxia [9] and A_2a_ receptors modulated rapid eye movements and breathing [10]. Both A_1_ and A_2a_ receptors regulated glutamate-induced depression of excitatory post synaptic potentials (EPSPs) [11]. Pharmacological studies can reveal which receptors adenosine acts at but do not give any information about the levels of adenosine that are available for signaling.

Techniques that directly measure adenosine release can be used to understand the amount of adenosine in the extracellular space. Early studies used radiometric labeling of adenosine coupled with HPLC analysis to examine the breakdown of ATP to adenosine [12]. Microdialysis coupled to HPLC was also used to measure adenosine increases [13]. These methods measured adenosine on the minute time scale but recently, electrochemical techniques have been developed that allow direct measurements on the second and even sub-second time scale. Fast, discrete release of adenosine has been characterized, which shows that adenosine exhibits characteristics of a neurotransmitter, as it is tightly regulated and cleared on a fast time scale. However, while adenosine has recently been reported in vesicles [14], there is currently no direct evidence that adenosine is released through exocytosis [15]. Enzyme biosensors specific for adenosine [16] have a response time of 2 s and were used to show that increases in adenosine occur within 2 min following ischemic events [17]. Fast-scan cyclic voltammetry (FSCV) at carbon-fiber microelectrodes directly measures adenosine on a sub-second time scale [18], [19], with a sampling rate of 10 times per second. FSCV has been used to study stimulated release *in vivo* [20], [21] and in brain slices [22], [23], [24]. These studies revealed that adenosine can be released and cleared in only a few seconds. However, the function of rapid adenosine release is still being elucidated. FSCV is the fastest method currently available for measuring adenosine changes and combined with pharmacology and electrophysiology, it has the capability of revealing how adenosine signals on a rapid time scale. In this review, we examine the fundamental principles of adenosine detection by FSCV, compare it to other measurement techniques, and highlight the biological applications and possible future studies that rapid measurements with FSCV may enable.

## 2 Adenosine Detection With Fast-scan Cyclic Voltammetry (FSCV)

**Fig. 1:**
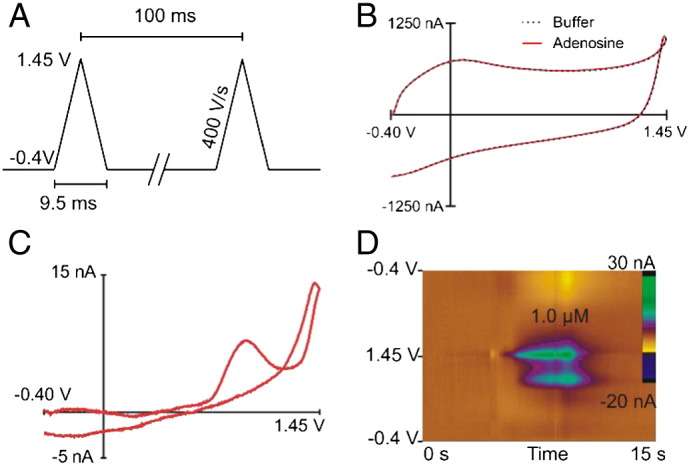
*Detection of adenosine with FSCV*. A) Applied potential waveform. The electrode is held at − 0.40 V, ramped up to a switching potential of 1.45 V and back at 400 V/s. The scan is repeated every 100 ms. B) The cyclic voltammogram (CV) is large due to the background charging current in the buffer (black dotted line) and the addition of 1.0 μM adenosine (red line). C) Subtracting out the background yields a background-subtracted CV of adenosine oxidation. The primary oxidation is observed at 1.4 V and the secondary oxidation at 1.0 V. The primary peak current is proportional to the concentration of adenosine detected at the electrode. D) False color plot of multiple background subtracted CVs. The *x*-axis is time, the *y*-axis is applied potential, and the color is current. This plot depicts an *in vitro* calibration experiment where the buffer is flowed by the electrode for 5 s, then 1.0 μM adenosine is flowed by for 5 s and finally buffer is flowed again. The large green oval in the center of the plot is the primary oxidation peak and the smaller green oval below is the secondary oxidation peak.

FSCV is an electrochemical technique that was developed to measure changes in electroactive neurotransmitters, especially dopamine [25]. To measure adenosine with FSCV, a triangular potential is applied scanning from − 0.40 V to 1.45 or 1.50 V and back versus a Ag/AgCl reference electrode at 400 V/s (Fig. 1A). The scan takes less than 10 ms and scans are repeated at 100 ms, which is the temporal resolution of the technique. The working electrode is a carbon-fiber microelectrode with a 7 μm diameter, which allows measurements in discrete brain regions. The fast scan rates cause a large background charging current (Fig. 1B) due to double layer charging at the electrode. The background current is stable over time and can be subtracted out from the signal [26], [27]. The result of subtracting the background current of the dashed line (buffer only) and the red line (buffer and adenosine) in Fig. 1B is a characteristic cyclic voltammogram for 1 μM adenosine (Fig. 1C).

**Scheme 1:**
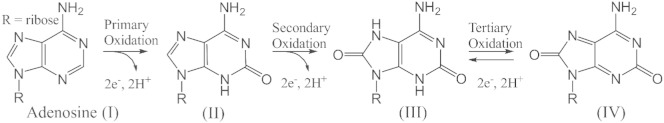
*Oxidation mechanism of adenosine*. Adenosine undergoes a three sequential, 2-electron oxidations. The first two oxidations are observed using FSCV and are irreversible. R is a ribose unit.

Adenosine is an electroactive molecule that can undergo a series of three, two-electron oxidations (Scheme 1) [28]. The initial oxidation of adenosine from product I to product II in Scheme 1 is observed at 1.4 V with FSCV. A secondary oxidation, from product II to product III, is detected at 1.0 V. The first two oxidation steps are irreversible and reduction peaks are not observed. The third oxidation in the scheme is seldom observed with FSCV at our carbon-fiber microelectrodes. Thus, the characteristic cyclic voltammogram (CV) for adenosine has two oxidation peaks, with the largest peak being near the switching potential at 1.4 V (Fig. 1C) [19].

Since many CVs are collected over time, it is useful to visualize multiple voltammograms simultaneously in false color plots (Fig. 1D). A vertical slice through the color plot at 7.5 s gives a CV of adenosine (Fig. 1C). The primary peak appears about a half second before the secondary peak on the color plot [29]. A horizontal slice through the color plot at 1.4 V shows how the oxidation current of adenosine changes against potential. With an appropriate calibration value, that current can be converted to concentration.

Traditionally, FSCV has been used to detect catecholamines such as dopamine [30] and norepinephrine [31] but it can also detect serotonin [32], histamine [33], and hydrogen peroxide [34]. Dopamine, serotonin, and norepinephrine have oxidation peaks around 0.6 V and reductions peaks between 0.2 V and − 0.2 V [35], [36] and the peaks do not interfere with adenosine detection. Hydrogen peroxide has a similar oxidation peak as adenosine at 1.2 V, but has no secondary peak, which distinguishes it from adenosine [34]. Histamine also has a similar oxidation potential as adenosine, however, the secondary peak potential is lower than that of adenosine [33].

The same electroactive adenine moiety is present in ATP and adenosine, so the electrochemical signatures of adenosine and ATP are similar. However, interferences can be minimized and adenosine distinguished from ATP with FSCV. Regular carbon-fiber microelectrodes were more sensitive for adenosine than for ATP when the applied waveform has a negative holding potential of − 0.4 V [19]. Electrodes coated with Nafion and carbon nanotubes were six-fold more sensitive for adenosine than for ATP [37]. Recently, our laboratory developed a sawhorse waveform that helped distinguish adenosine and ATP [38]. The altered waveform was more sensitive for adenosine and gave distinct signals for adenosine over ATP; however there was still some overlap between the two molecules. To positively identify adenosine release *in vivo*, pharmacological manipulations were used [20] and the identity of the analyte was verified by independent techniques, such as biosensors [39]. These approaches were similar to those used to distinguish dopamine from other catecholamines [40] and verified that adenosine was monitored by FSCV.

## 3 Comparison of Adenosine Detection With FSCV and Other Methods

### 3.1 Comparison of Microdialysis and FSCV

Microdialysis is one of the most general techniques for monitoring neurochemical changes. A microdialysis probe is inserted into the brain and artificial cerebral spinal fluid is pumped through the probe. The probe is semi-permeable allowing molecules to diffuse and be collected in the dialysate. The aliquot is usually analyzed with HPLC and one advantage is that many species can be measured from the same sample [41]. For example, adenosine was quantified and separated from hypoxanthine and uric acid [42]. The limit of detection for adenosine was as low as 5 nM using microdialysis [43]. Microdialysis monitoring has revealed that adenosine is released during ischemia and built up in 15 min [44]. Microdialysis samples were collected for 5 to 10 min, so the time scale of the measurement is slower than FSCV. Microdialysis is good for studying slower changes in basal levels of adenosine while FSCV is better at detecting rapid fluctuations in extracellular adenosine.

### 3.2 Comparison of Electrophysiology and FSCV

Electrophysiology studies monitor the firing of neurons and can be used to examine the effects of adenosine release. For example, adenosine depressed EPSPs and thus decreased post-synaptic neurotransmission [45]. Electrophysiology studies found that endogenous adenosine release in hippocampal slices was activity dependent and acted *via* A_1_ receptors [46].

Electrophysiology studies look at downstream effects of adenosine on cell firing, while electrochemical methods directly measure adenosine release. Thus, the two methods are complementary. Electrophysiology measurements are on the millisecond time scale and were used to demonstrate that adenosine acted at A_1_ receptors on a 1–2 second time scale [46]. FSCV has confirmed that adenosine release can last only a few seconds [20] and showed rapid signaling of adenosine in the brain. FSCV and electrophysiology studies have been combined at the same microelectrode to measure dopamine [47] and similar studies could be performed in the future to monitor the amount of adenosine release and its effect on neuronal firing.

### 3.3 Comparison of FSCV and Adenosine Biosensors

**Fig. 2:**
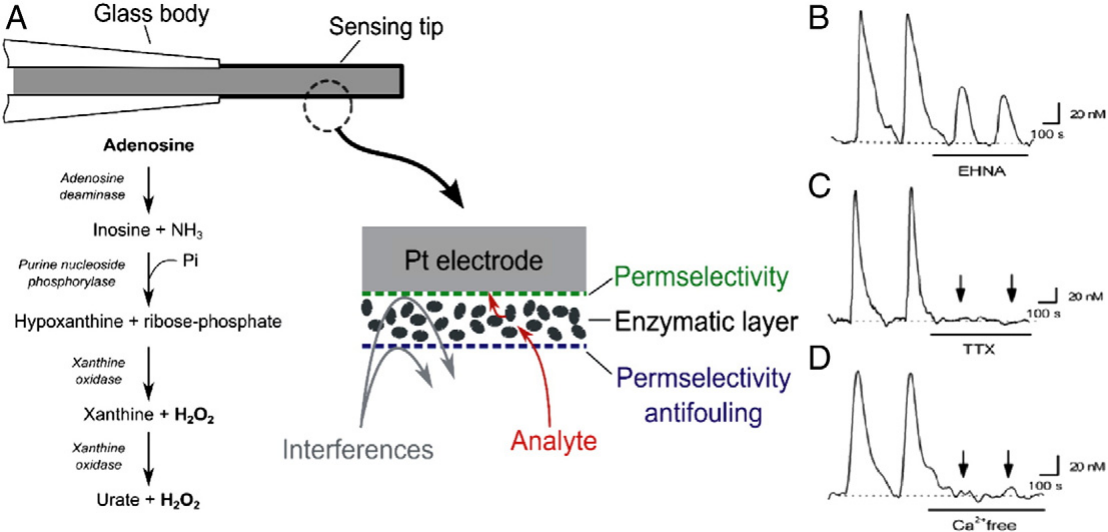
*Biosensor detection of adenosine*. Left: A) Schematic of adenosine biosensor. The platinum electrode is coated with a permselective layer to prevent fouling and an enzymatic layer that breaks down adenosine to hydrogen peroxide. Right: Evoked adenosine release in brain slices. B) EHNA, an adenosine deaminase inhibitor, lowers evoked adenosine release. C) Tetrodotoxin (TTX), which inhibits action potentials, completely eliminates stimulated adenosine release. D) Ca^2 +^ free buffer abolishes evoked adenosine release.

Another method for detecting adenosine is using amperometric biosensors, which were developed by the Dale group [16]. Adenosine biosensors directly measure adenosine at platinum electrodes coated with enzymes that metabolize adenosine to hydrogen peroxide, which is detected amperometrically at + 0.5 V [48]. Adenosine is broken down to inosine, then to hypoxanthine, then to xanthine, urate, and hydrogen peroxide *via* adenosine deaminase, purine nucleoside phosphorylase, and xanthine oxidase, respectively (Fig. 2A) [49]. Although the biosensors are held at + 0.5 V, the multiple polymer layers may act as a barrier to prevent electroactive species from oxidizing [48], while still allowing for detection of H_2_O_2_ without slowing response time [49]. An identical null sensor which contains no adenosine deaminase is placed next to the biosensors to distinguish adenosine from any interferents, particularly downstream metabolites. Subtracting out the null sensor signal from the adenosine biosensor signal gives a specific response for adenosine. The limit of detection for adenosine biosensors is 12 nM and the rise time is about 2 s, which allows measurements of adenosine release on the second time scale [48]. Fig. 2 shows evoked adenosine release measured by biosensors. Adenosine release was decreased by EHNA (erythro-9-(2-hydroxy-3-nonyl)adenine) hydrochloride, which inhibits adenosine deaminase (Fig. 2B); tetrodotoxin, a blocker of action potentials (Fig. 2C); and removal of Ca^2 +^ (Fig. 2D), which blocks activity dependent release [50].

Biosensors have been used to examine the mechanism and function of adenosine release, particularly in brain slices. In hippocampal slices, 5–10 min of hypoxia gradually increased adenosine over the ischemic event [17]. Similarly, adenosine released during hypoxia was activity dependent and not a downstream breakdown product of ATP [50], [51]. Enzyme biosensors were also used to measure electrically stimulated [52] and glutamate-induced adenosine release [53]. These studies revealed that adenosine was released on a time scale of seconds to minutes following oxygen deprivation, electrical stimulation, or glutamate application.

FSCV and amperometric biosensors are both electrochemical techniques that measure adenosine directly. The limit of detection for adenosine at carbon-fiber microelectrodes is comparable to biosensors [48], in the 15 nM range. FSCV at carbon-fiber microelectrodes has better temporal resolution than biosensors, with rise times for FSCV being only a few hundred milliseconds. Typically, adenosine biosensors are platinum electrodes with diameters of 25 and 50 μm while carbon-fiber microelectrodes are only 7 μm. Smaller electrodes cause less tissue damage [54]. Adenosine release measured with FSCV lasts only a couple of seconds while the release measured with biosensors is usually longer, on the order of a minute. Biosensors require a null sensor to screen out metabolites, while metabolites such as inosine are electrochemically inactive at carbon-fiber electrodes using FSCV [19]. However, biosensors are able to measure basal changes in adenosine over a minute to hour time scale, while FSCV cannot observe basal shifts because the signal is background subtracted. With both techniques, evoked adenosine release is activity dependent on the rapid time scale [23], [50]. Biosensors for ATP have also been developed and can be used to distinguish adenosine and ATP signals [55].

### 3.4 Advantages and Disadvantages of FSCV

There are several advantages of FSCV for detection of adenosine. The high sensitivity for adenosine allows measurements in the physiologically relevant range of receptor affinities [56]. Adenosine is directly detected, as opposed to measuring downstream effects, which is beneficial for determining mechanisms of release that may account for adenosine signaling [57]. Another benefit of FSCV with carbon-fiber microelectrodes is the small size of the electrodes (7 μm), which are less invasive than microdialysis probes of 250–500 μm [58]. Carbon-fiber microelectrodes are also cheap and easy to make, as opposed to biosensors which are usually purchased from a commercial source and are more expensive.

The biggest advantage of FSCV is the time resolution of the measurements, 100 ms, which is the fastest method for measuring adenosine. Adenine nucleotides undergo conversion to adenosine within 200 ms, suggesting a need to detect adenosine on a fast time scale [59]. Biosensors take around 2 s to respond to a change in adenosine and thus are not fast enough to characterize many of the spontaneous adenosine transient signals that last only 3 s on average [29]. The consequence of fast scanning is a large background current, so local changes in concentration are typically only measured over a 90 second window due to background subtraction [60]. Using analog background subtraction with principal component analysis, changes in dopamine concentrations have been quantified over a 30 minute period [26], however this method has not been tested with adenosine. A disadvantage of FSCV is that it cannot measure basal concentrations of adenosine because it requires background subtraction. However, FSCV is the best method for measuring rapid changes and when combined with pharmacology can be a powerful tool to understand transient adenosine signaling.

## 4 Biological Studies of Adenosine With FSCV

### 4.1 Electrically-stimulated Adenosine Release

**Fig. 3:**
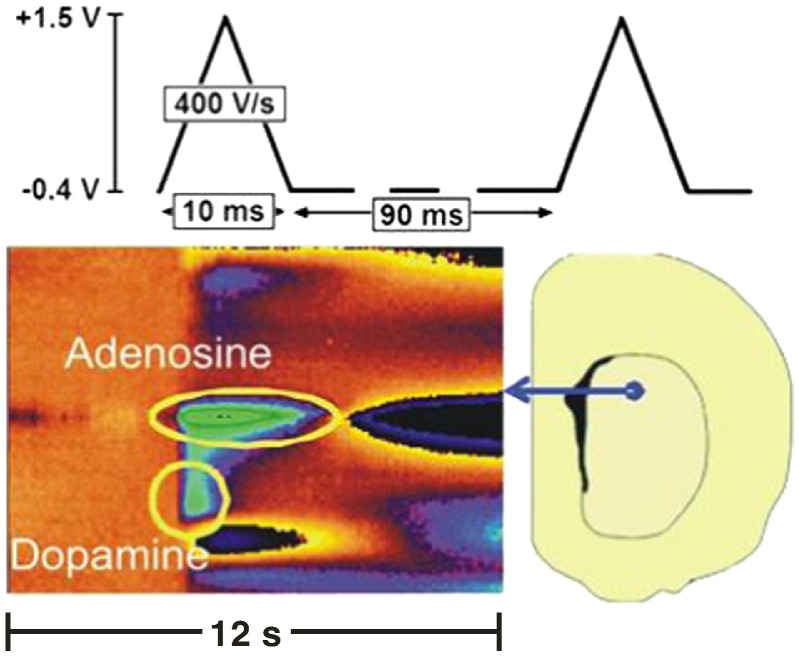
*Electrically stimulated adenosine in slices using FSCV*. The applied waveform is shown above the color plot. Electrically stimulated adenosine and dopamine are shown on the false color plot in yellow circles. The dopamine and adenosine are cleared within 2 and 5 s of stimulation, respectively. A diagram of the location of electrode implantation in the caudate–putamen is shown to the right.

Using FSCV, stimulated adenosine was characterized in anesthetized animals [20] and brain slices [22]. Electrically-stimulated release was first characterized *in vivo* in the caudate–putamen after dopamine neurons in the medial forebrain bundle were stimulated [20]. Both the evoked dopamine release and adenosine release occurred immediately. Fig. 3 shows a false color plot in brain slices of stimulated dopamine release and adenosine release being cleared in 2 and 5 s, respectively [23]. On average, the peak evoked adenosine was 0.94 μM and lasted 15 s, *in vivo* [20]. Pharmacological experiments demonstrated that propentofylline, an inhibitor of adenosine transport, decreased stimulated release while ABT 702, an adenosine kinase inhibitor, increased evoked release. Stimulated adenosine release increased oxygen flow within 5 s at the electrode, demonstrating that adenosine can control cerebral blood flow on a rapid time scale. A_1_ receptors self-regulated adenosine release [21]; the A_1_ agonist, CPA, decreased evoked adenosine while the A_1_ antagonist, DPCPX, increased adenosine release rapidly.

FSCV was also used to characterize stimulated adenosine release in brain slices. Two different pulse trains, low and high frequency, were compared in rat striatal slices [23]. The high frequency stimulations (5 pulses, 60 Hz) evoked more adenosine than the low frequency stimulations (5 pulses, 10 Hz). Blocking nucleoside transporters did not change evoked adenosine concentration so stimulated adenosine was not released through equilibrative transporters. Stimulated adenosine release varied in different brain regions [24], with higher levels evoked in the caudate–putamen and nucleus accumbens compared to the cortex and hippocampus. While basal levels also differ in distinctive regions [61], there was little trend between evoked levels of adenosine [24] and previously measured basal levels [62], [63].

The mechanism of stimulated adenosine release was examined on a rapid time scale. Evoked adenosine release was activity dependent because Ca^2 +^ chelation or application of tetrodotoxin (TTX) almost completely reduced the signal [24]. Thus, rapid adenosine release was likely due to exocytosis of adenosine, exocytosis of its precursor ATP, or the downstream result of activity-dependent neurotransmitter release [57]. NMDA and AMPA antagonists decreased the release of adenosine in the caudate–putamen and nucleus accumbens, showing the dependence of adenosine release on ionotropic glutamate receptors [24]. The effect of blocking ATP metabolism varied by brain region. AOPCP and ARL 67156, inhibitors of ATP metabolism, had no effect in the caudate–putamen, but decreased adenosine in the nucleus accumbens, hippocampus, and cortex. Thus, there are at least two mechanisms of stimulated adenosine release: breakdown of extracellular nucleotides and downstream effects of ionotropic glutamate receptors, but these mechanisms differ by brain region.

### 4.2 Adenosine Release During Deep Brain Stimulation Probe Implantation

**Fig. 4:**
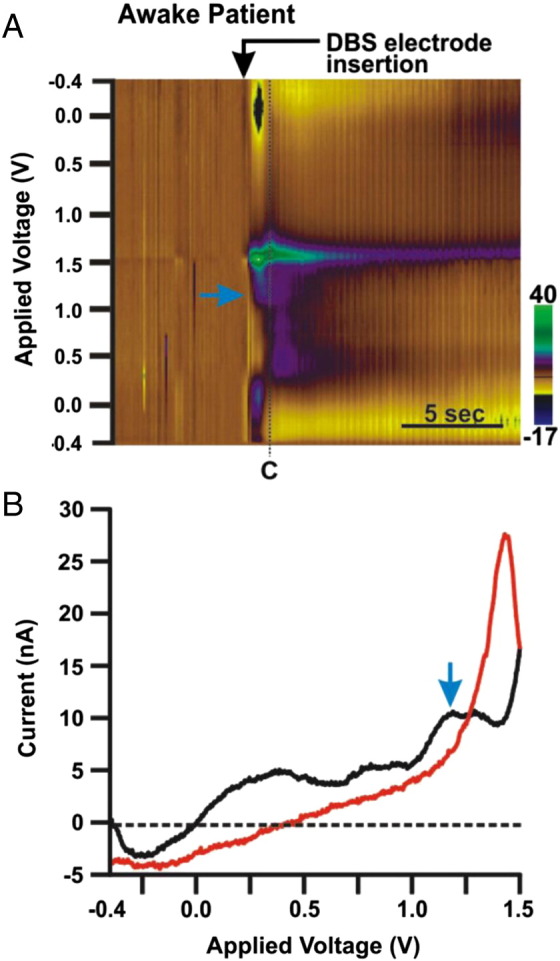
*Adenosine release by DBS probe implantation*. A) False color plot of DBS electrode implantation in the human patient. The black arrow denotes the insertion of the electrode and the blue arrow shows the immediate release of adenosine. B) A cyclic voltammogram of adenosine shows the characteristic primary and secondary oxidation of adenosine at 1.4 V and 1.0 V, respectively.

FSCV is being pioneered for human clinical trials in order to monitor neurotransmitters during deep brain stimulation (DBS) [64]. DBS is used to treat tremors but a phenomenon known as the microthalamotomy effect has been observed where simply implanting a probe in the thalamus reduces tremors without applying an electrical stimulation [65]. Release of adenosine during probe implantation was hypothesized to be important for this effect. Using FSCV, the implantation of a carbon-fiber electrode into the thalamus was shown to release adenosine within seconds (Fig. 4) [66]. Adenosine release was also observed concurrently with tremor arrest [67]. Thus, FSCV in clinical trials is a valuable technique for understanding the release of adenosine on a rapid time scale during microelectrode implantation and DBS treatment.

### 4.3 Mechanically Stimulated Adenosine Release

Our laboratory characterized the effect of small mechanical stimulations by moving an electrode, or a pipette near an electrode, a small distance. Adenosine transiently increased after mechanical stimulation and was cleared within 20 s [39]. Mechanical release of adenosine was observed *in vivo* and in brain slices and was not due to cell death or tissue damage [39]. Mechanically-stimulated adenosine decreased following the application of EDTA (to complex Ca^2 +^) and TTX, showing that the release was activity dependent. Blocking the nucleoside transporter with NBTI did not diminish the signal, proving that the release was not through transporters. Mechanically-stimulated release was partially blocked by POM-1, which inhibits the breakdown of ATP, showing that some of the release was from ATP metabolism. The rapid release of adenosine following electrode implantation or brain damage could be neuroprotective.

### 4.4 Spontaneous, Transient Adenosine Release

Spontaneous, transient adenosine release was discovered in spinal cord slices of mice using FSCV [68]. The duration of adenosine release was only 1.5 s and the average concentration was 0.53 μM. This rapid, transient release could not have been detected with microdialysis or biosensors, as only FSCV has sub-second temporal resolution. The frequency of transient release was low, with events occurring once every 3 min and was significantly reduced when Ca^2 +^ was removed from the buffer, suggesting activity dependent release of adenosine. Knockout mice lacking NT5E and CD73, enzymes that convert AMP to adenosine, also had a lower frequency of transients. A third enzyme, tissue non-specific alkaline phosphatase (TNAP), was later found to inhibit the rapid conversion of applied AMP to adenosine in the double knockout mice with FSCV [69]. The results suggested that adenosine transients were formed from rapidly hydrolyzed AMP; however the inhibition of TNAP has not been tested for spontaneous, transient adenosine release.

**Fig. 5:**
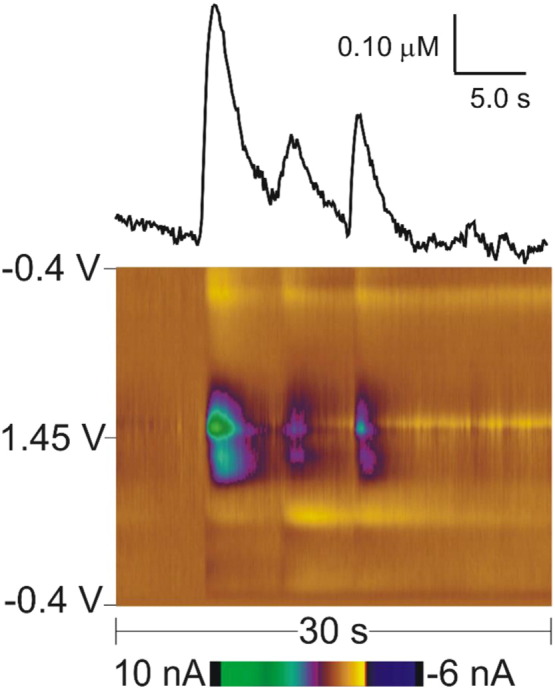
*Spontaneous transient adenosine release with FSCV*. A false color plot shows three spontaneous release events of adenosine in a 20 second window. The concentration vs time trace above the color plot shows the three discrete events of adenosine.

Spontaneous, transient adenosine release was also characterized *in vivo* in anesthetized rats [29]. No stimulation was applied and adenosine release was observed that lasted only 3 s. Fig. 5 shows a false color plot of three transients that occur within 20 s of each other. FSCV has the temporal resolution to detect three distinct adenosine events in this short time frame. The average adenosine concentration was 0.17 μM in the caudate–putamen and 0.19 μM in the prefrontal cortex, however transients as large as 2.5 μM were observed. The release was random and events occurred on average about once every 3 min. A_1_ receptors modulated the frequency of transient adenosine release, but the mechanism of regulation has not been elucidated. Interestingly, comparing the mice and rat studies, the characteristics of spontaneous transient adenosine release were similar even though they were measured in two different rodent species and in two different regions [29], [68]. This suggests that spontaneous, transient adenosine release is a conserved signaling pathway across species and regions.

## 5 Future Challenges

### 5.1 Determining the Mechanism and Location of Release

The biggest finding from FSCV research on adenosine has been the discovery of a mode of signaling that lasts only a few seconds. The sub-second temporal resolution of FSCV was needed to understand the temporal dynamics of rapid release. Electrically-stimulated release, mechanically-stimulated release, and spontaneous, transient adenosine release all occur on this rapid time scale. Transient adenosine release can be regulated by A_1_ receptors and partially by ionotropic glutamate receptors [24]. FSCV has facilitated a better understanding of a rapid signaling role for adenosine but questions still persist.

One of the remaining questions is about the mechanism of adenosine release that leads to this rapid signaling. Extensive research from multiple groups has concluded that rapid adenosine release is activity dependent and not released through nucleoside transporters [23], [24], [39], [57], [68], [70]. A key question is whether adenosine is directly released or is a breakdown product of ATP. In some studies, a portion of the rapid adenosine released is due to ATP metabolism, but in most studies there is also a portion of adenosine that is not from ATP. Because there are multiple enzymes that breakdown ATP [69], [71] and some of the inhibitors of these enzymes are inefficient [72], it is difficult to rule out ATP as the sole source of adenosine. However, adenosine was recently discovered in vesicles [14], so future work should examine the possibility of direct release of adenosine from exocytosis versus breakdown of exocytotically released ATP.

The exact cells that release adenosine on this rapid time scale have not been distinguished and identifying the cells might also aid in understanding the mechanism of release. ATP is released as a neuronal co-transmitter with dopamine, serotonin, GABA, and glutamate [73] and adenosine release could be due to metabolism of ATP from those neurons. Using Ca^2 +^ imaging techniques, adenosine release has been identified from astrocytes [74] and adenosine can be cleared by glial cells as well [75]. FSCV can be used to measure exocytosis directly from single cells, so future studies could examine adenosine release from neurons and astrocytes to better understand the mechanism and location of adenosine release.

### 5.2 Understanding the Function of Rapid Adenosine Release

**Fig. 6:**
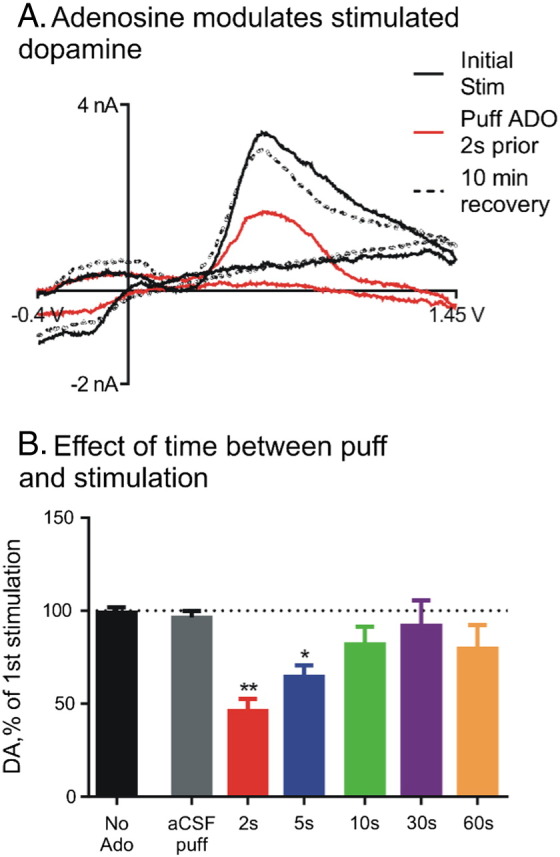
*Transient modulation of stimulated dopamine release with FSCV*. (A) Cyclic voltammograms for stimulated dopamine release initially (black line), 2 s after adenosine application (red line), and 10 min after adenosine application (dashed line). (B) Stimulated dopamine after application of adenosine over different times. Evoked dopamine significantly decreased if adenosine was applied 2 or 5 s before stimulation.

When a new signaling mode of a molecule is identified, it is important to characterize the function of that signaling. Transient adenosine likely acts locally, as the distance a molecule such as adenosine could diffuse in a couple of seconds is only 10–20 μm [76]. Adenosine is known to modulate neurotransmission in the brain and so an obvious hypothesis is that transient adenosine regulates neurotransmission on a rapid time scale. Recently, our laboratory identified that transient adenosine release rapidly modulated phasic dopamine release. When adenosine was transiently applied less than 10 s before dopamine was electrically evoked, the stimulated dopamine concentration was reduced by 50% (Fig. 6) [77]. This inhibitory effect was mediated by A_1_ receptors and only occurred while adenosine was present in the extracellular space. Thus, rapid adenosine is able to modulate neurotransmission in discrete locations. Adenosine also modulated oxygen concentrations in the brain [20], suggesting that transient adenosine release can locally modulate oxygen and blood flow. Future studies could explore the extent to which transient adenosine modulates other neurotransmitters.

FSCV should be useful in the future for understanding the role of rapid adenosine release in diseases. For example, during epileptic events, adenosine decreased excitatory glutamate release and hyperpolarized neurons, which is desirable during hyper-excitatory seizures [78]. FSCV was used to detect adenosine release during epilepsy and seizures in pigs and was safe in human experiments [79]. Adenosine was released prior to seizure termination and remained elevated following the epileptic event, which confirms the idea that adenosine is involved in seizure expiration [80]. FSCV has rapid time resolution so it could be used to monitor adenosine during the rapid onset of seizures. FSCV could help distinguish whether adenosine release is constant during all seizures and determine if adenosine is released before or after the onset of seizures.

### 5.3 Final Conclusions

FSCV allows adenosine detection on a sub-second time scale and has been used to uncover a rapid mode of adenosine signaling that lasts only a few seconds. Transient adenosine release can be measured after electrical or mechanical stimulations. It can also be observed spontaneously in brain slices and *in vivo*, when no stimulation is applied. This transient release modulates phasic dopamine and oxygen flow, which indicates that rapid adenosine release has a fast neuromodulatory role. Future studies will use FSCV in conjunction with pharmacology, electrophysiology, and biosensors to discover more about the mechanism and function of this mode of rapid adenosine signaling.

## Figures and Tables

Data adapted from Nguyen et al. [29].

Data adapted with permission from Dale [49], [50] and Springer Science and Business Media.

Figure reprinted from [23] Copyright 2010 American Chemical Society.

Figure adapted from Lee's group [67] Copyright Elsevier (2012).

Figure adapted from Nguyen et al. [29].

Figure adapted from Ross and Venton [77] with permission from Wiley Company.
